# Protective Effects of Hydrogen Treatment Against High Glucose-Induced Oxidative Stress and Apoptosis *via* Inhibition of the AGEs/RAGE/NF-κB Signaling Pathway in Skin Cells

**DOI:** 10.2174/0118715303369584241231141001

**Published:** 2025-01-08

**Authors:** Pan Yu, Nan Hong, Qiong Wu, Zhipeng Zhao

**Affiliations:** 1 Department of Burn and Plastic Surgery, Jinling Hospital, Affiliated Hospital of Medical School, Nanjing University, Nanjing, China

**Keywords:** Diabetes, hydrogen, oxidative stress, advanced glycation end products, receptor for advanced glycation end products, metabolic disorder

## Abstract

**Background:**

Diabetic wounds are major clinical challenges, often complicated by oxidative stress and free radical generation. Hydrogen (H_2_), a selective antioxidant, offers potential as a therapeutic agent for chronic diabetic wounds. However, its precise mechanisms remain underexplored.

**Objective:**

This study aimed to investigate the protective effects of H_2_ on high glucose-induced oxidative damage and apoptosis in human skin cells.

**Methods:**

HaCaT keratinocytes and HSF fibroblasts were treated with high glucose or AGEs. Cell viability, oxidative stress markers, inflammatory cytokines, and apoptosis were analyzed. AGEs/RAGE/NF-κB signaling was evaluated via Western blot.

**Results:**

H_2_ treatment significantly reduced ROS, MDA, IL-1β, and TNF-α levels, while enhancing SOD and GSH activity. It also inhibited AGEs/RAGE/NF-κB signaling and apoptosis.

**Conclusion:**

Hydrogen therapy protects against oxidative stress and inflammation induced by high glucose or AGEs, offering potential as an adjunctive treatment for diabetic wound healing.

## 1 INTRODUCTION

Diabetes Mellitus (DM) has emerged as a leading threat to human health amid the economic development of many countries. This multi-factorial metabolic disorder is characterized by chronic hyperglycemia, which results from either a defect in insulin secretion, insulin resistance, or a combination of both. The pathogenesis of diabetes remains unknown, contributing to a wide range of complications [1, 2]. Among these complications, individuals with diabetes are particularly prone to recurrent skin conditions and non-healing wounds, which frequently lead to the development of chronic ulcers. In severe cases, these ulcers can progress to a stage where amputation becomes necessary, causing significant distress to both patients and their families.

The persistent elevation and fluctuations in blood glucose levels in diabetic individuals lead to a progressive accumulation of advanced glycosylation end products (AGEs). AGEs are formed through a non-enzymatic reaction between the amino groups of proteins and the aldehyde groups of glucose, and they are notably resistant to degradation by acids and enzymes [3, 4]. The accumulation of AGEs has significant biological implications, as they influence fibroblast proliferation [5-7]. Additionally, during the process of non-enzymatic glycosylation, AGEs generate substantial amounts of reactive oxygen species (ROS), which further contribute to cellular damage [8]. Aminoguanidine has been identified as a potential inhibitor of AGE formation; it reacts with methylglyoxal, an α-dicarbonyl compound involved in AGEs synthesis, thereby preventing or reducing AGE accumulation [9].

Excessive ROS production can disrupt the redox balance and inhibit collagen synthesis [10]. During the process of ROS generation, the superoxide anion (O_2_^-^) is the primary species produced [11, 12]. Numerous studies have [13-15] demonstrated that levels of ROS and O_2_^-^ are significantly elevated in diabetes, which adversely affects the progression of wound healing associated with diabetes. The enzymatic conversion of O_2_^-^ by SOD leads to the formation of hydroxyl radicals (-OH), which rapidly react with nitric oxide (NO) to form peroxynitrite anion (ONOO-) [16]. This reaction results in rapid lipid peroxidation and the nitration of various amino acids, such as tyrosine, ultimately causing damage to neurological functions. Consequently, diabetic patients are more susceptible to purulent infections, including boils and abscesses, as well as fungal infections and various ulcers that are difficult to heal [9].

Historically, hydrogen (H_2_) was considered a physiologically inert gas. However, its potential biological significance gained attention in 2007 when Ohsawa *et al*. [17] demonstrated its ability to significantly scavenge free radicals, particularly in the context of ischemia-reperfusion (I/R) brain injury, through the inhalation of 2% H_2_. Further research revealed that when H_2_ was dissolved in a liquid medium, it can selectively neutralize -OH and ONOO-. Sun *et al*. [18] pioneered the development of Hydrogen-rich saline, demonstrating its therapeutic efficacy through intraperitoneal administration in various conditions, such as acute pancreatitis [18], acute lung injury [19], severe acute carbon monoxide (CO) poisoning [20], and cardiac ischemia-reperfusion (I/R) injury [21]. Building on these findings, subsequent studies have revealed that treatment with hydrogen-rich water (HW) can improve insulin levels [22], reduce oxidative stress and inflammation, and alleviate dysfunction in the liver, kidneys, and spleen in hyperglycemic rat models [23]. Moreover, the administration of H_2_ is characterized by its low toxicity, potent antioxidant properties, and ease of application, making it a promising candidate for therapeutic use [24].

In our previous studies [25], we developed a murine wound model using streptozotocin-induced diabetic mice, administering hydrogen-rich saline via intraperitoneal injection. The findings revealed that H_2_ significantly enhanced wound healing in diabetic mice, by reducing the ROS production at the wound site, and decreasing AGEs levels within the wound. In this study, human skin fibroblasts (HSF) and keratinocytes (HaCaT) were cultured under conditions of elevated glucose or AGEs to evaluate the effects of H_2_ on cellular proliferation and apoptosis. The study assessed changes in cell apoptosis, levels of oxidative products, inflammatory mediators, and AGEs/RAGE/NF-κB signaling pathways. These results elucidate the protective role of H_2_ against cellular damage induced by high glucose, thereby providing a robust foundation for its potential clinical application in enhancing diabetic wound healing. This study integrates molecular and therapeutic perspectives on metabolic and immune dysfunction, aligning with the journal's focus on interventions targeting endocrine-metabolic- immune disorders.

## 2 MATERIALS AND METHODS

### 2.1 Materials

Human immortalized HaCaT and HSF cells were procured from the American Type Culture Collection (ATCC, Manassas, VA, United States). Dulbecco's modified eagle medium (DMEM) and fetal bovine serum (FBS) were obtained from Hyclone (Beijing, China). Methyl thiazolyl tetrazolium (MTT) was acquired from Sigma-Aldrich Co. (St. Louis, MO, USA). Dimethyl sulfoxide (DMSO) and the annexin V fluorescein isothiocyanate (FITC)/propidium iodide (PI) kit were sourced from Invitrogen (Thermo Fisher Scientific, Waltham, MA, USA). A ROS assay kit was obtained from Beyotime (Shanghai, China). SOD kit, MDA kit, and GSH kit were purchased from Nanjing Jiancheng Bioengineering Institute (Nanjing, China). Enzyme-linked immunosorbent assay (ELISA) kits for 8-hydroxy-2'-deoxyguanosine (8-OHdG) and 3-nitrotyrosine (3-NT) were acquired from R&D Systems (Minneapolis, MN, USA), while ELISA kits for interleukin-1 beta (IL-1β) and tumor necrosis factor-alpha (TNF-α) were obtained from Elabscience (Wuhan, China). A bicinchoninic acid (BCA) protein quantification kit was purchased from Beyotime (Shanghai, China). Enhanced chemiluminescence (ECL) kit was sourced from Millipore (MA, USA). Antibodies for glyceraldehyde 3-phosphate dehydrogenase (β-actin (ab115777), a receptor for advanced glycation end products (RAGE) (ab216329), nuclear factor kappa-light-chain-enhancer of activated B cells (NF-κB/p65) (ab207297), an inhibitor of kappa B (IκB)α (phospho S32) (ab92700), and IκBα (ab32518) were obtained from Abcam (Cambridge, U.K.)

### 2.2 Cell Culture and Treatment

Previous studies [17, 26] utilized the AMS-H-01 H_2_ nebulizer (Asclepius, Shanghai, China) to generate a mixed gas composed of H_2_ and oxygen (O_2_). Subsequently, this mixture was further combined with nitrogen (N_2_) using a three-way connector. The concentration of hydrogen in the final gas mixture was precisely calibrated to 60%, 40%, and 20% (vol/vol).

HaCaT and HSF cells were cultured in DMEM supplemented with 10% FBS, 100 units/mL of penicillin, and 100 µg/mL of streptomycin at 37°C in a humidified atmosphere containing 5% carbon dioxide (CO_2_). For experimental procedures, the cells were seeded into 6-well plates at a density of 1.0×10^5^ cells/mL, exposed to high glucose or AGEs, and treated with either H_2_ or aminoguanidine, respectively, before undergoing further assays.

### 2.3 Cell Viability Assay

HaCaT and HSF cells were uniformly seeded in a 96-well plate with 3000 cells/well. To investigate the time- course and dose-dependent effects of H_2_ on cellular responses, the cells were subjected to varying concentrations of H_2_ (20%, 40%, and 60%) for 24 h, 48 h, and 72 h at 37°C. Additionally, to examine the impact of advanced glycation end-products (AGEs) on cellular responses, the cells were incubated with different concentrations of AGEs (100 μg/mL, 200 μg/mL, 400 μg/mL, and 600 μg/mL) at 37°C for 48 and 72 hours. In subsequent experiments, the cells were exposed to high glucose or AGEs and treated with either H_2_ or aminoguanidine, respectively, to further elucidate the cellular response mechanisms. Cell viability was assessed using routine MTT assay.

### 2.4 Cell Apoptosis Analysis Using Flow Cytometry

Cell apoptosis was analyzed *via* flow cytometry with an Annexin V-FITC/PI apoptosis detection kit. Briefly, HaCaT and HSF cells were incubated with FITC-labeled Annexin V and PI for 15 min at room temperature. Subsequently, the cells were washed with PBS, centrifuged at 1500 rpm for 5 min, and resuspended in a binding buffer for analysis. Fluorescent signals were measured using a fluorescence-activated cell sorting (FACS) flow cytometer (BD Calibur, USA), and cell cycle phases were analyzed with CellQuest Pro software.

### 2.5 Reactive Oxygen Species (ROS) Assay

HaCaT and HSF cells were cultured in 48-well plates at a seeding density of 6×10^3^ cells per well. The experimental groups were subjected to various treatments, after which ROS probe 2',7'-dichlorofluorescin diacetate (DCFH-DA) was introduced to the cell culture medium at a dilution of 1:1,000. The cells were then incubated at 37°C for 30 min and subsequently washed three times with PBS. Intracellular ROS levels were assessed using a fluorescence microscope at a magnification of ×200 (Leica Microsystems GmbH).

### 2.6 SOD, GSH-PX Activity, and MDA Detection

The levels of SOD, glutathione peroxidase (GSH-PX) activity, and MDA were quantified using specific assay kits designed for each parameter. Initially, action buffers were added to the supernatants of the cell samples, which were then incubated at 37°C. Optical densities were subsequently measured using a visible spectrophotometer at wavelengths of 550 nm, 412 nm, and 532 nm. These measurements allowed for the evaluation of SOD and GSH-PX activities, as well as the quantification of MDA levels.

### 2.7 ELISA

The inflammatory factors, TNF-α and IL-1β, as well as biomarkers of cellular oxidants, including 8-OHdG and 3-NT, were quantified using ELISA techniques. In summary, the culture supernatants from both the control group and the high-glucose-treated group were collected, and the expression levels of TNF-α, IL-1β, 8-OHdG, and 3-NT were assessed in accordance with the manufacturer's instructions.

### 2.8 Western Blot

HaCaT and HSF cells were cultured under various treatment conditions, as previously described, in six-well plates. All samples were collected and lysed using RIPA lysis buffer supplemented with protease inhibitors, phosphatase inhibitors, and phenylmethylsulfonyl fluoride (PMSF). The total protein concentration in the samples was quantified using a BCA protein quantification kit. Following denaturation, forty micrograms of protein were separated by sodium dodecyl sulfate-polyacrylamide gel electrophoresis (SDS-PAGE) and subsequently transferred to a polyvinylidene fluoride (PVDF) membrane (Millipore, Bedford, MA, USA). The membrane was then blocked with bovine serum albumin (BSA) for 1 h at room temperature, after which it was incubated with primary antibodies against AGEs (1:1000), RAGE (1:1000), NF-κB/p65 (1:1000), p-IκB-α (1:1000), and IκB-α (1:1000) at 4 °C overnight, followed by incubation with corresponding horseradish peroxidase (HRP)-conjugated secondary antibodies at room temperature for 1 h. The protein bands were visualized using an ECL kit (Millipore, MA, USA).

### 2.9 Statistical Analysis

Data were collected from at least three independent experiments, and the results were presented as the mean ± SD. The experimental data were analyzed using SPSS18.0 and GraphPad Prism 9. One-way ANOVA, two-way ANOVA, and Tukey’s multiple comparisons test were used to analyze the differences among multiple groups. *P*< 0.05 was considered as statistical significance.

## 3 RESULTS

### 3.1 H_2_ Treatment Promotes HG-Inhibited Cell Viability and Decreases Apoptosis

**Fig. (1):**
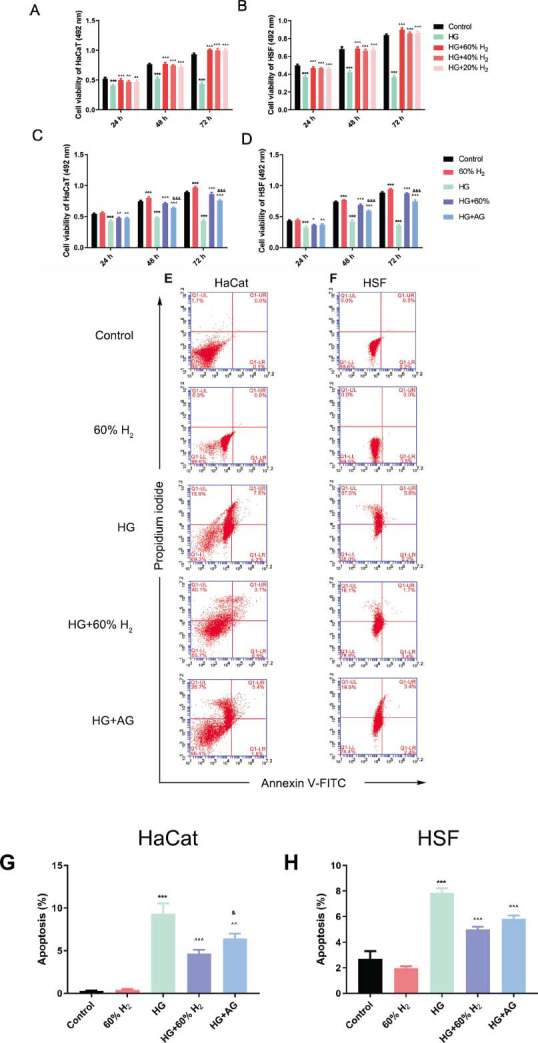
H_2_ treatment enhances cell viability and decreases apoptosis in HG-exposed HaCaT cells and HSF cells. Relative cell activity measured using MTT method at different concentrations and times after treatment in (**A**) HaCaT cells and (**B**) HSF cells; Cell viability analyzed using MTT assay in (**C**) HaCaT cells and (**D**) HSF cells; (**E**-**H**) Flow cytometric analysis of apoptosis using FITC annexin V/PI double staining in (**E**, **G**) HaCaT cells and (**F**, **H**) HSF cells. (Note: Dot plots illustrate necrotic cells (Q1: annexin V-FITC−/PI+), late apoptotic cells (Q2: annexin V-FITC+/PI+), live cells (Q3: annexin V-FITC−/PI−), and early apoptotic cells (Q4: annexin V-FITC+/PI−). All data are expressed as means±SD. Statistical significance: ***P > 0.001 compared to the control group; ^*P* > 0.05, ^^*P* > 0.01, ^^^*P* > 0.001 compared to HG group; & *P* > 0.05, &&& *P* > 0.001 compared to HG+60% H_2_ group.

To assess the effect of H_2_ treatment on cellular activity, this study investigated variations in cell activity across different concentrations and time intervals. Exposure to 50 mmol/L glucose resulted in a significant inhibition of HaCaT and HSF cell proliferation over time (*P* < 0.05). However, subsequent H_2_ treatment markedly restored cell growth, with the most pronounced effect observed at a 60% H_2_ concentration (*P* < 0.05). As a result, a 60% H_2_ concentration was selected for further investigation (Figs. **1A** and **1B**). Elevated glucose levels are known to lead to the accumulation of AGEs [27, 28]. The experimental results demonstrated that a 60% H_2_ concentration at 48 and 72 h enhanced cell activity more effectively than aminoguanidine, an inhibitor of AGE formation, at 500 μmol/L [29] (*P* < 0.05) (Figs. **1C** and **1D**). Consequently, the duration of H_2_ treatment in subsequent experiments was established at 48 h.

Annexin V-FITC and PI staining were employed to evaluate the impact of H_2_ treatment on cell apoptosis. The apoptosis analysis revealed that exposure to 50 mmol/L glucose, in the presence of 60% H_2_, significantly reduced high-glucose-induced apoptosis (*P* < 0.05), as indicated by an increase in Annexin V-FITC-positive cells. These findings collectively suggest that H_2_ treatment not only improves cell viability under elevated glucose conditions but also decreases the rate of apoptosis (Figs. **1E**-**H**).

### 3.2 H_2_ Treatment Attenuates HG-Mediated Oxidative Stress and Inflammation

**Fig. (2):**
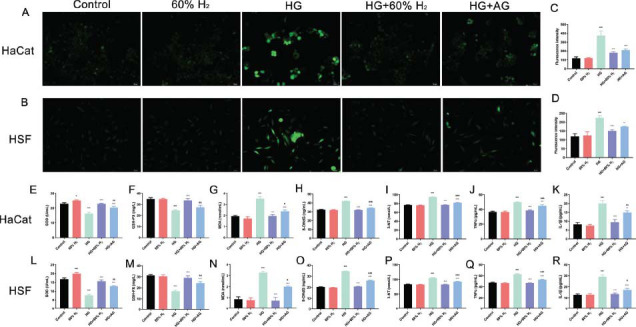
H_2_ treatment decreases HG-mediated oxidative stress and inflammation HG-exposed in HaCaT cells and HSF cells. ROS measured using DCFH-DA surface fluorescence in (**A**) HaCaT  cells and (**B**) HSF  cells; Quantification of DCFH-DA intensity REFLECTING ROS in (**C**) HaCaT  cells and (**D**) HSF  cells; Levels of oxidative stress markers (SOD, GSH-PX, MDA, 8-OHdG 3-NT) and pro-inflammatory cytokines (TNF-α and IL-1β) in (**E**-**K**) HaCaT cells and (**L**-**R**) HSF cells. (**Note:** *P > 0.05, ***P > 0.001 compared to the control group; ^^*P* > 0.01, ^^^*P* > 0.001 compared to HG group; & *P* > 0.05, && *P* > 0.01, &&&*P* > 0.001 compared to HG+60% H_2_ group).

Elevated glucose levels are widely recognized to enhance the production of intracellular reactive oxygen species (ROS), which play a critical role in triggering apoptosis under both physiological and pathological conditions [30]. To investigate whether hydrogen (H_2_) treatment can inhibit intracellular ROS generation in HaCaT and HSF cells, a DCFH- DA assay was employed in this study. The results demonstrated that exposure to high-glucose conditions led to a significant increase in ROS production (*P* < 0.05). However, both H_2_ treatment and aminoguanidine effectively reduced ROS levels compared to the high-glucose group (*P* < 0.05). Notably, no statistically significant difference was observed between the H_2_ treatment group and the aminoguanidine group (*P* ≥ 0.05), suggesting comparable efficacy in suppressing ROS production. (Figs. **2** and **2A**-**D**).

The research further investigated the levels of oxidative stress and inflammatory markers, focusing on SOD, GSH-PX, MDA, 8-OHdG, 3-NT, TNF-α and IL-1β. The results indicated that the levels of MDA, 8-OHdG, 3-NT, TNF-α and IL-1β were elevated (*P* > 0.05), while the levels of SOD and GSH-PX were reduced in HaCaT and HSF cells exposed to 50 mmol/L glucose (*P* > 0.05). Treatment with H_2_ resulted in a reduction of MDA, 8-OHdG, 3-NT, TNF-α and IL-1β levels (*P* > 0.05), alongside an increase in SOD and GSH-PX levels (*P* > 0.05).

### 3.3 H_2_ Treatment Attenuates HG-Inhibited AGEs/RAGE/NF-κB Signaling Pathway

**Fig. (3):**
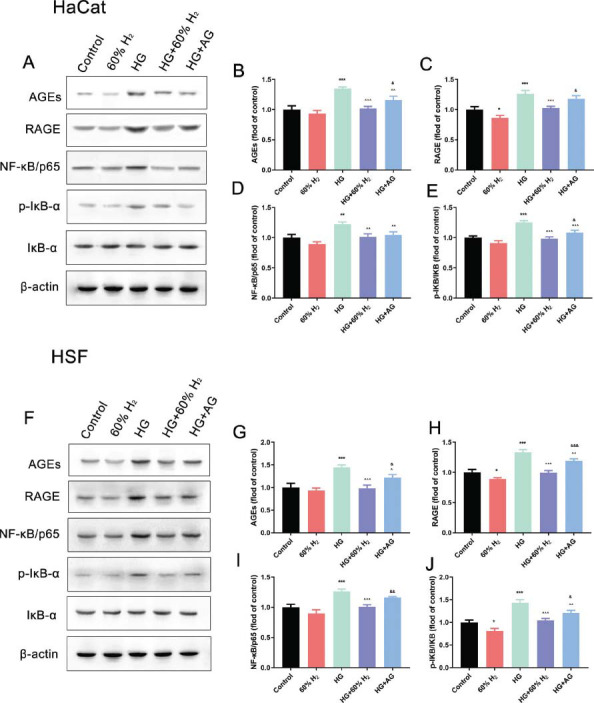
H_2_ treatment inhibits AGEs/RAGE/NF-κB signaling pathway in HG-exposed HaCaT cells and HSF cells. Protein expression levels of AGEs, RAGE, NF-κB/p65, p-IκB-α, and IκB-α detected via western blot analysis in (**A**) HaCaT cells and (**F**) HSF cells; Quantification of (**B**) AGEs, (**C**) RAGE, (**D**) NF-κB/p65 and (**E**) p-IκB-α in HaCaT cells. Quantification of (**G**) AGEs, (**H**) RAGE, (**I**) NF-κB/p65, and (**J**) p-IκB-α in HSF cells. (**Note:** *P > 0.05, **P > 0.01, ***P > 0.001 compared to the control group; ^*P* > 0.05, ^^*P* > 0.01, ^^^*P* > 0.001 compared to HG group; & *P* > 0.05, && *P* > 0.01, &&& *P* > 0.001 compared to HG+60% H_2_ group).

Elevated glucose levels are a significant factor in the impaired wound healing observed in diabetic patients, primarily due to the accumulation of advanced glycation end-products (AGEs) and their interaction with the receptor for advanced glycation end-products (RAGE) on the cell surface. This interaction is a critical mechanism that exacerbates oxidative stress, further hindering the healing process. To elucidate this mechanism, the expression of proteins associated with the AGEs/RAGE/NF-κB signaling pathway was examined and validated using Western blot analysis. Compared to the control group, the levels of AGEs, RAGE, NF-κB/p65, and phosphorylated IκB-α (p-IκB-α)/IκB-α ratio were significantly elevated following the exposure of HaCaT and HSF cells to high glucose (*P* < 0.05). Conversely, the protein expression of AGEs, RAGE, and NF-κB/p65 decreased (*P* < 0.05) after treatment with 60% H_2_, and the phosphorylation level of IκB-α also diminished (*P* < 0.05). The addition of aminoguanidine produced a similar effect on the cells, although its effect was slightly less pronounced than that of H_2_ (*P* ≥ 0.05). Collectively, these results suggest that high glucose levels promote the activation of the AGEs/RAGE/NF-κB signaling pathway, while H_2_ therapy may inhibit this effect (Fig. **3**).

### 3.4 H_2_ Treatment Increases AGEs-Inhibited Cell Apoptosis

**Fig. (4):**
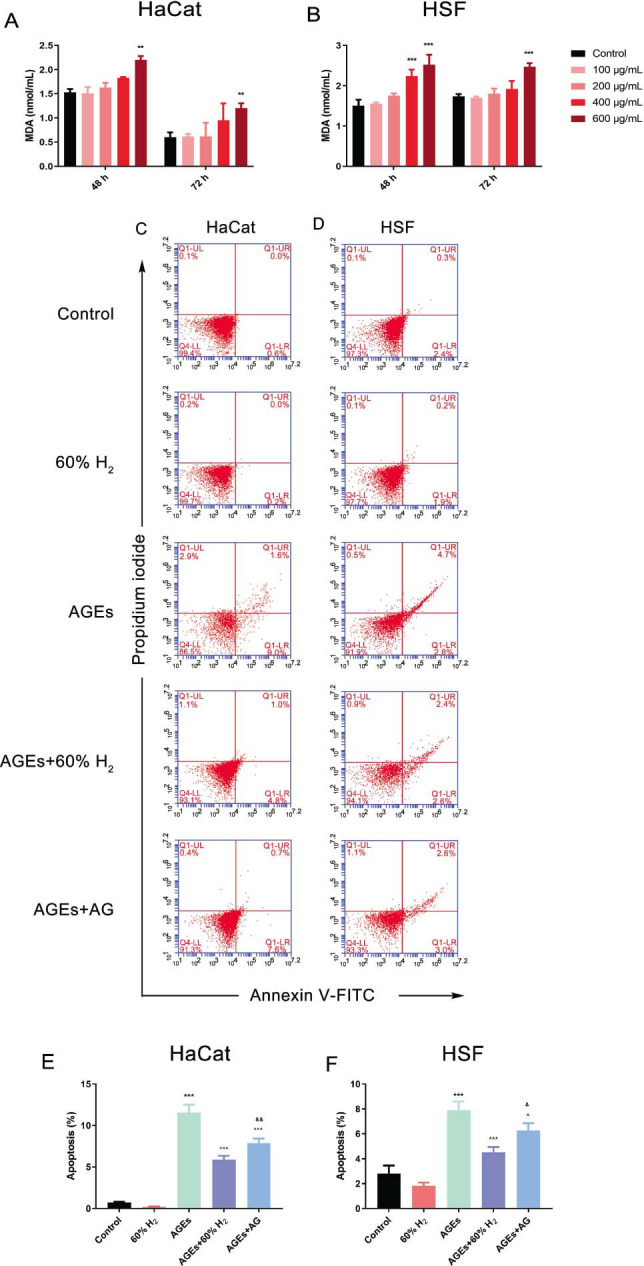
H_2_ treatment increases AGEs-induced HaCaT cells and HSF cells. Levels of MDA in (**A**) HaCaT cells and (**B**) HSF cells following treatment with different concentrations of AGEs at 48 h and 72 h; Flow cytometric FITC annexin V/PI double staining analysis of apoptosis in (**C**, **E**) HaCaT cells and (**D**, **F**) HSF cells. (Note: Dot plots illustrate necrotic cells (Q1: annexin V-FITC−/PI+), late apoptotic cells (Q2: annexin V-FITC+/PI+), live cells (Q3: annexin V-FITC−/PI−), and early apoptotic cells (Q4: annexin V-FITC+/PI−). All data are expressed as means±SD. Statistical significance: **P > 0.01, ***P > 0.001 compared to the control group; ^*P* > 0.05, ^^^*P* > 0.001 compared to AGEs group; &*P* > 0.05, &&*P* > 0.01 compared to AGEs+60% H_2_ group).

To explore the effect of H_2_ treatment on reducing oxidative stress in high-glucose-induced HaCaT and HSF cells via the AGEs/RAGE/NF-κB signaling pathway, this study quantified malondialdehyde (MDA) levels using an MDA reagent kit. Cells were exposed to varying concentrations of AGEs for 48 and 72 hours. The results demonstrated a positive correlation between MDA levels and both the concentration of AGEs and the duration of exposure. Specifically, MDA levels increased significantly after 72 hours of treatment with 400 μg/mL of AGEs (*P* < 0.05). Based on this statistically significant finding, the condition of 400 μg/mL AGEs for 72 hours was selected for subsequent experimental procedures (Figs. **4A** and **4B**).

Apoptosis levels in HaCaT and HSF cells induced by AGEs (400 μg/mL for 72 h) were assessed using flow cytometry following treatments with 60% H_2_ and aminoguanidine. AGE exposure in the presence of 60% H_2_ significantly reduced AGE-induced apoptosis, as indicated by a decrease in Annexin V-FITC-positive cells (*P* < 0.05) (Figs. **4C**-**F**). These results demonstrated that while AGEs effectively induce apoptosis in HaCaT and HSF cells, H_2_ therapy has the potential to mitigate these apoptotic effects.

### 3.5 H_2_ Treatment Decreases AGEs-Induced Oxidative Stress and Inflammation

**Fig. (5):**
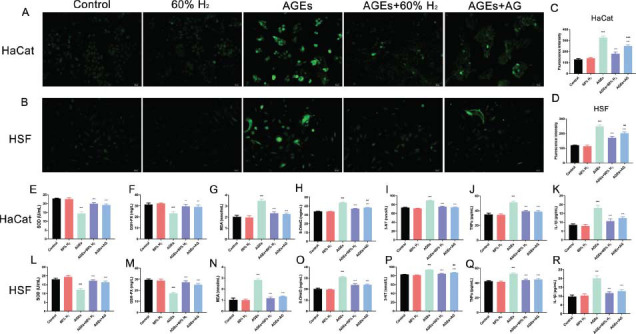
H_2_ treatment decreases AGEs-induced oxidative stress and inflammation in HaCaT cells and HSF cells. ROS measured using DCFH-DA surface fluorescence in (**A**) HaCaT  cells and (**B**) HSF  cells; Quantification of DCFH-DA fluorescence intensity reflecting ROS in (**C**) HaCaT  cells and (**D**) HSF  cells; Levels of oxidative stress markers (SOD, GSH-PX, MDA, 8-OHdG 3-NT) and pro-inflammatory cytokines (TNF-α and IL-1β) in (**E**-**K**) HaCaT cells and (**L**-**R**) HSF cells. (**Note:** ***P > 0.001 compared to the control group; ^^*P* > 0.01, ^^^*P* > 0.001 compared to AGEs group; && *P* > 0.01, &&& *P* > 0.001 compared to AGEs+60% H_2_ group).

Exposure to 400 μg/mL of AGEs for a duration of 72 h resulted in a significant increase in the release of ROS in HaCaT and HSF cells (*P* > 0.05). This was accompanied by elevated levels of MDA, 8-OHdG, 3-NT, TNF-α and IL-1β (*P* > 0.05). Concurrently, a reduction in SOD and GSH-PX levels was observed (*P* > 0.05). Treatment with H2 was found to reverse these effects more effectively than aminoguanidine, leading to a decrease in intracellular ROS, MDA, 8-OHdG, 3-NT, TNF-α and IL-1β levels (*P* > 0.05), while simultaneously increasing SOD and GSH-PX levels (*P* > 0.05) (Figs. **5**).

### 3.6 H_2_ Treatment Attenuates AGEs-Induced AGEs/RAGE/NF-κB Signaling Pathway

**Fig. (6):**
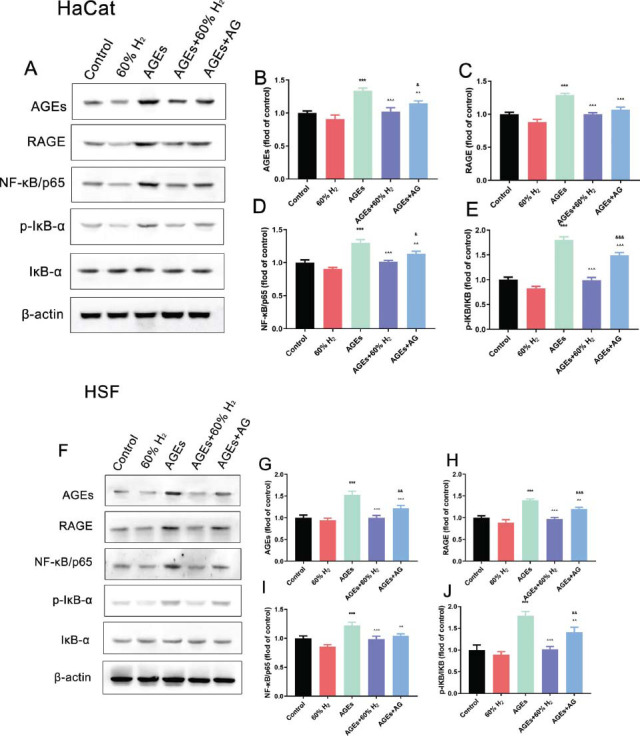
H_2_ treatment inhibits AGEs/RAGE/NF-κB signaling pathway in HG-exposed HaCaT cells and HSF cells. Protein expression levels of AGEs, RAGE, NF-κB/p65, p-IκB-α, and IκB-α detected *via* western blot analysis in (**A**) HaCaT cells and (**F**) HSF cells; Quantification of (**B**) AGEs, (**C**) RAGE, (**D**) NF-κB/p65, and (**E**) p-IκB-α in HaCaT cells. Quantification of (**G**) AGEs, (**H**) RAGE, (**I**) NF-κB/p65, and (**J**) p-IκB-α in HSF cells. (Note: ***P > 0.001 compared to the control group; ^^*P* > 0.01, ^^^*P* > 0.001 compared to AGEs group; &*P* > 0.05, &&*P* > 0.01, &&&*P* > 0.001 compared to AGEs+60% H_2_ group).

In the final analysis, this study researched the protein expressions related to the AGEs/RAGE/NF-κB signaling pathway. After the treatment of AGEs, the protein level ratios of AGEs, RAGE, NF-κB/p65, and p-IκB-α/IκB-α were increased significantly compared to those in the control group (*P* < 0.05). On the contrary, the protein expressions related to AGEs, RAGE, and NF-κB/p65 were downregulated in H_2_-treated groups (*P* < 0.05); meanwhile, the phosphorylation degree of IκB-α was reduced (*P* < 0.05). Aminoguanidine also exhibited a protective influence on the cells, which was marginally less compared to H_2_ treatment (*P*≥0.05) (Fig. **6**). In conclusion, H_2_ treatment may decrease the AGEs/RAGE/NF-κB signaling pathway induced via AGEs.

## 4 DISCUSSION

With the improvement in living standards, there has been a significant increase in the prevalence of diabetes, leading to a substantial number of patients suffering from chronic wounds or diabetic foot ulcers and resulting in an amputation rate of 15% [31]. A critical factor impeding wound healing in individuals with diabetes is excessive oxidative stress, which persists despite the effective management of blood glucose levels through glucose-lowering treatments [32]. H_2_, the smallest molecule in nature, has demonstrated various biological effects, including antioxidant properties, inhibition of apoptosis, and regulation of autophagy. Consequently, H_2_ therapy is currently being explored as an emerging therapeutic strategy. This study aims to elucidate a novel mechanism by which H_2_ therapy alleviates high-glucose-induced oxidative stress in HSF and HaCaT through the down-regulation of AGEs/RAGE/NF-κB signaling pathway.

Current clinical antioxidants have not demonstrated significant improvements in the healing of diabetic wounds, and many are associated with adverse reactions that limit their long-term use. Identifying effective antioxidants with minimal side effects could significantly enhance the healing process of diabetic wounds. Hydrogen (H_2_), the simplest gaseous molecule in nature, has the ability to specifically neutralize -OH and ONOO-, thereby reducing oxidative stress [16]. Research suggests that H_2_ may function not only through its antioxidant properties but also via alternative mechanisms, potentially acting as a novel signaling gas molecule [33]. Numerous studies have demonstrated the therapeutic efficacy of H_2_ in various conditions, including diabetic stroke [34], sepsis [35], pancreatitis [17], brain injury [36, 37], and constipation [38]. Currently, a significant number of clinical studies are being conducted both domestically and internationally to explore the application of H_2_ in the treatment of COVID-19 [39], cardiopulmonary bypass [40], and diabetic nephropathy [41]. In our previous study [25], H_2_ was found to enhance wound healing in diabetic murine models; however, the precise mechanisms underlying this effect remain unclear. Due to its small molecular size, H_2_ readily permeates cellular membranes to exert its function. Various methods have been employed to administer H_2_ to cells, such as introducng pre-mixed H_2_-containing gas into sealed environments and using H_2_-enriched media. Nevertheless, these methods fail to maintain a stable H_2_ concentration in cells over time. Given its safety profile and ease of application, hydrogen therapy represents a promising, non-toxic adjunct for improving diabetic wound outcomes. Therefore, this study developed a new H_2_ incubator that allows for adjustable H_2_ levels, facilitating the investigation of optimal concentration and duration for H_2_ treatment in cells.

Advanced glycation end-products (AGEs) are formed through non-enzymatic glycation reactions, and their accumulation is particularly problematic in individuals with diabetes. This accumulation contributes to oxidative stress by increasing the production of free radicals, which complicates the wound-healing process [42]. The present study demonstrates that elevated levels of glucose or AGEs result in reduced cell viability and increased intracellular ROS compared to control groups. Furthermore, treatment with 60% H_2_ was found to decrease cell death and lower ROS levels. These findings align with recent studies suggesting that H_2_ treatment effectively mitigates oxidative stress [43], particularly in the context of diabetes [44, 45].

Elevated blood glucose levels contribute to increased cellular oxidative stress and a reduction in antioxidant capacity [46]. Superoxide dismutase (SOD) plays a crucial role as a scavenger of oxygen radicals, serving as an indicator of the body's free radical scavenging ability, which is essential for maintaining the balance of reactive oxygen species (ROS). Glutathione peroxidase (GSH), the main non-enzymatic antioxidant, works in conjunction with SOD to mitigate damage caused by high glucose [47]. Malondialdehyde (MDA), a product of lipid peroxidation, rises when cellular antioxidant defenses are inadequate. Previous studies have reported that MDA content, a biomarker of oxidative stress, is significantly elevated in the skin granulation tissue of diabetic rats with ulcers [48]. Consequently, we assessed AGEs concentrations by examining the increase in MDA content induced by AGEs. Furthermore, 8-hydroxy-2'-deoxyguanosine (8-OHdG) and 3-nitrotyrosine (3-NT) are recognized markers of oxidative DNA damage and are used to evaluate DNA damage resulting from various factors [49]. Consistent with Xu *et al*. [50], our findings demonstrate that H_2_ treatment significantly enhances SOD and GSH levels while concurrently reducing MDA, 8-OHdG, and 3-NT levels following exposure to high glucose or AGEs.

During the synthesis of AGEs, a significant amount of ROS radicals is produced, which further promotes the non-enzymatic glycosylation reaction. This process affects cellular function and induces cytotoxic effects. In the later stages of glycosylation, irreversible AGEs are formed, exemplified by glycosylated hemoglobin, a stable product resulting from the non-enzymatic reaction between glucose and hemoglobin [51]. The formation of AGEs generates numerous free radicals, initiating oxidation processes that accelerate AGE production, thereby creating a vicious cycle. AGEs can directly affect cellular and tissue functions by binding to RAGEs, leading to pathological changes within the body [28, 52]. RAGE is expressed in HSF and HaCaT cells, with low levels present in normal tissues and blood vessels. However, elevated levels of AGE in elderly and diabetic patients contribute to the high expression of RAGEs in these individuals [53]. The binding of AGEs to RAGE triggers the release of tissue factors and adhesion molecules, activating nuclear factor kappa B (NF-κB), which results in the expression of inflammatory mediators, such as interleukin-1 (IL-1) and tumor necrosis factor-alpha (TNF-α), ultimately causing tissue damage. A critical step in NF-κB activation involves the phosphorylation and degradation of its inhibitor, IκB-α [54]. Previous research has indicated that molecular hydrogen mediates neurorestorative effects after stroke in diabetic rats by inhibiting NF-κB phosphorylation and reducing inflammation [34]. Our results further highlight that H_2_ treatment attenuates the production of inflammatory substances and cellular damage by inhibiting the activation of the AGE/RAGE/NF-κB pathway.

In conclusion, this study demonstrates that H_2_ can effectively reduce the accumulation of AGEs and mitigate skin cellular damage induced by diabetes without causing toxicity. These findings highlight the potential of H_2_ in modulating cellular-oxidative stress, suggesting its possible application as an adjunctive therapy for chronic diabetic skin ulcers. However, further research is needed to explore the long-term effects of hydrogen therapy, particularly when combined with other therapeutic agents, to enhance the efficacy of diabetic wound healing *in vivo*. Such investigations may uncover synergistic benefits, which could lead to optimized treatment protocols and improved outcomes for patients with diabetic wounds.

## CONCLUSION

In summary, the experimental results provide compelling evidence that H_2_ treatment exhibits significant protective effects against high-glucose-induced cellular damage in both HaCaT and HSF cells. Specifically, H_2_ administration effectively attenuates oxidative stress and reduces cellular apoptosis. Mechanistic investigations revealed that this cytoprotective action is predominantly mediated through the downregulation of the AGEs/RAGE/NF-?B signaling pathway. These findings collectively suggest that H_2_ intervention holds promising therapeutic potential for both the prevention and treatment of high-glucose-mediated cellular injury, warranting further investigation as a clinical treatment modality.

## AUTHORS’ CONTRIBUTIONS

The authors confirm their contribution to the paper: study conception and design: P.Y.; data collection: N.H., Q.W., and Z.Z.; analysis and interpretation of results: N.H., Q.W., and Z.Z.; draft manuscript: P.Y. All authors reviewed the results and approved the final version of the manuscript.

## Data Availability

All data are available from the corresponding author [PY] upon reasonable request.

## LIST OF ABBREVIATIONS

ROS: Reactive Oxygen Species
HW: Hydrogen-Rich Water
HSF: Human Skin Fibroblasts
FBS: Fetal Bovine Serum
AGEs: Advanced Glycation Endproducts
FACS: Fluorescence-Activated Cell Sorting
GSH-PX: Glutathione Peroxidase
PMSF: Phenylmethylsulfonyl Fluoride
